# Navigating advanced renal cell carcinoma in the era of artificial intelligence

**DOI:** 10.1186/s40644-025-00835-7

**Published:** 2025-02-18

**Authors:** Elie J. Najem, Mohd Javed S. Shaikh, Atul B. Shinagare, Katherine M. Krajewski

**Affiliations:** 1 Department of Imaging, Dana-Farber Cancer Institute, Boston, MA USA; 2 Department of Radiology, Brigham and Women’s Hospital, Boston, MA USA

## Abstract

**Background:**

Research has helped to better understand renal cell carcinoma and enhance management of patients with locally advanced and metastatic disease. More recently, artificial intelligence has emerged as a powerful tool in cancer research, particularly in oncologic imaging.

**Body:**

Despite promising results of artificial intelligence in renal cell carcinoma research, most investigations have focused on localized disease, while relatively fewer studies have targeted advanced and metastatic disease. This paper summarizes major artificial intelligence advances focusing mostly on their potential clinical value from initial staging and identification of high-risk features to predicting response to treatment in advanced renal cell carcinoma, while addressing major limitations in the development of some models and highlighting new avenues for future research.

**Conclusion:**

Artificial intelligence-enabled models have a great potential in improving clinical practice in the diagnosis and management of advanced renal cell carcinoma, particularly when developed from both clinicopathologic and radiologic data.

## Introduction

Artificial intelligence (AI) has recently emerged as a powerful tool in cancer research with exceptional capability in identifying complex patterns and extracting quantifiable information from large datasets. This allows for the transition of data interpretation from a subjective, qualitative process to one that is objective and reproducible. Furthermore, it facilitates the integration of diverse data sources, such as radiologic images, genomics, pathology, electronic health records, and social networks, into comprehensive diagnostic systems, ultimately assisting physicians in evidence-based clinical decision making [1].

**Fig. 1:**
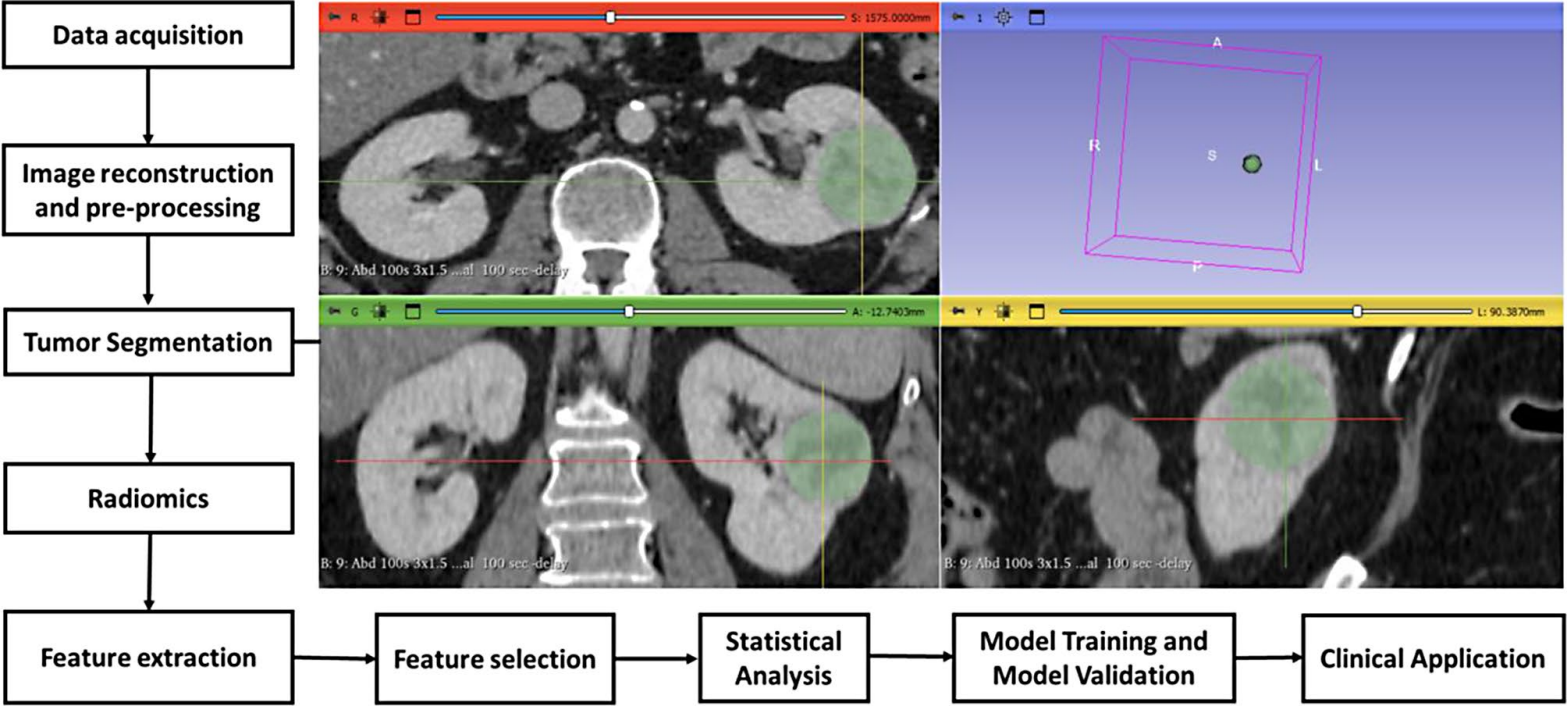
Schematic pipeline of Radiomics workflow. Following image acquisition and tumor segmentation, the radiomic features are extracted. The features selected are those that most effectively represent variability in the data or optimize a specific predictive model. High-level statistical modeling including machine learning and deep learning techniques are then employed for disease classification, patient grouping and tailored risk assessment

In diagnostic radiology, the definition of AI remains somewhat ambiguous, encompassing a vast spectrum of domains such as machine learning (ML), deep learning (DL), neural networks (NNs), artificial neural networks (ANNs), convolutional neural networks (CNNs), recurrent neural networks (RNNs), natural language processing (NLPs) [2], radiomics [3] (Fig. 1), and radiogenomics [4] (Table 1). Each approach or combination thereof offers distinct benefits and limitations. Generally, more advanced techniques enhance diagnostic accuracy and adaptability but necessitate larger curated datasets and longer development and training times. In practice, the primary objective of these diverse AI methodologies in diagnostic radiology is to develop algorithms that support radiologists in effectively conveying imaging findings and ultimately improving patient care [5, 6].

**Table 1:** This table defines multiple AI categories along with their key applications

Category	Description	Key applications
Machine Learning (ML)	Predicts patterns in data using mathematical algorithms. Includes methods like deep learning, logistic regression, and neural network architecture.	Automates cancer detection and diagnosis.
Deep Learning (DL)	Utilizes multilayer neural networks inspired by the brain. Extracts features, analyzes large datasets, and enhances cancer diagnosis and treatment.	Early cancer detection, diagnosis, grading, molecular characterization, predicting outcomes, personalized treatment, clinical trials, and drug discovery.
Neural Networks (NNs)	Complex ML systems including ANN, MLP, RNN, and CNN, designed to process intricate datasets and improve over time through adaptive learning.	Diagnosis, treatment, and outcome prediction in complex clinical scenarios.
Artificial Neural Networks (ANNs)	Computational systems modeled after the human nervous system, capable of adaptive learning and analyzing relationships among clinical, biological, and pathological variables.	Widely used in cancer diagnosis and predictive modeling.
Convolutional Neural Networks (CNNs)	Specialized in processing large image datasets and extracting features using convolutional filters. Adapted for non-image data like genomic vectors.	Cancer imaging analysis, genomic data processing, and feature extraction.
Recurrent Neural Networks (RNNs)	Processes sequential data by leveraging hidden state vectors for context-based predictions.	Analysis of time-series data in healthcare.
Natural Language Processing (NLP)	Transforms unstructured text into structured data for analysis by applying techniques like named entity recognition and relationship extraction.	Clinical data organization, extracting key information, and building structured databases.
Radiomics	Extracts a large number of quantitative features from medical images using advanced computational algorithms.	Tumor characterization, prediction of treatment response, patient outcomes, and guiding precision oncology.
Radiogenomics	Integrates imaging features with genomic data to uncover relationships between radiologic phenotypes and genetic markers.	Understanding tumor biology, predicting therapeutic responses, and enabling personalized treatment strategies.

In cancer imaging, AI has been studied in three main clinical domains: tumor detection, characterization, and monitoring. Specifically, within the realm of kidney cancer imaging, AI algorithms are commonly designed to non-invasively address four key questions: the type of renal cancer, its stage, its grade, and the likelihood of metastatic disease. In recent decades, there has been a significant increase in renal mass detection and a contemporaneous rise in AI as a powerful tool in cancer imaging research. Therefore, it is not surprising that most available studies in the literature primarily focus on the diagnosis and grading of renal tumors. Beyond renal cell carcinoma (RCC) subtype, stage and grade, there are opportunities to use imaging to optimize risk stratification and clinical decision making over the care continuum [7].

Locally advanced RCC is characterized by at least one of the following features: spread into peri-renal or peri-pelvic fat, extension into major veins, invasion of the adrenal gland, spread to adjacent retroperitoneal nodes or penetration of Gerota’s fascia [7, 8]. With locally advanced features carrying prognostic and therapeutic implications, particularly venous wall invasion, renal and the inferior vena cava tumor thrombi, early detection of these features is essential [7]. High risk features of RCC identified include tumor stage 2 with nuclear grade 4 or sarcomatoid differentiation, tumor stage 3 or higher, regional node metastases, or oligometastatic RCC resected to no evidence of disease [9]. Recent AI studies address some unmet clinical needs, in identifying locally advanced disease pre-operatively, predicting high risk features or distant metastatic disease, and assessing tumor response to treatment [7].

In this review paper, we provide a multidisciplinary assessment of various applications of AI in locally advanced and metastatic renal cancer, focusing mainly on the current performance of imaging-based AI solutions.

## Initial RCC staging and high-risk features

The TNM system for renal cell carcinoma was most recently described in the 8th edition of the American Joint Committee on Cancer (AJCC) staging manual. Tumor size and local extension of the primary tumor determine the T category, involvement of local (retroperitoneal) lymph nodes determines the N category and the presence of distant metastases determines the M category [9, 10]. Renal biopsy, previously underutilized due to concerns about safety and sampling errors, is increasingly being recognized as a valuable tool in the diagnosis and management of renal masses. Advances in biopsy techniques and pathological interpretation have not only expanded its applications, including diagnosing indeterminate masses, evaluating candidates for active surveillance, and guiding targeted therapies for metastatic disease but also decreased rates of complications (0.3–5.3%), and minimized risks, such as biopsy tract seeding. Nonetheless, its accuracy remains highly variable ranging from 38 to 100%, which could add an additional layer of complexity in the accurate diagnosis, staging and treatment of RCC [10].

While surgery remains the mainstay treatment option for localized RCC, up to 30% of patients develop recurrent or advanced disease post-nephrectomy [11]. Features of locally advanced disease have been associated with increased risk of recurrence and poor prognosis [12]. Several clinical trials have examined the feasibility of neoadjuvant [13–16] and adjuvant [17–20] therapies in locally advanced and high-risk patients, respectively. High risk patients may benefit from neoadjuvant or adjuvant treatment, while low- and intermediate-risk patients with non-metastatic RCC likely do not need it and should not be subjected to the associated risks [21], highlighting the need for refined risk stratification and optimized staging.

### Prediction of locally advanced RCC

**Fig. 2:**
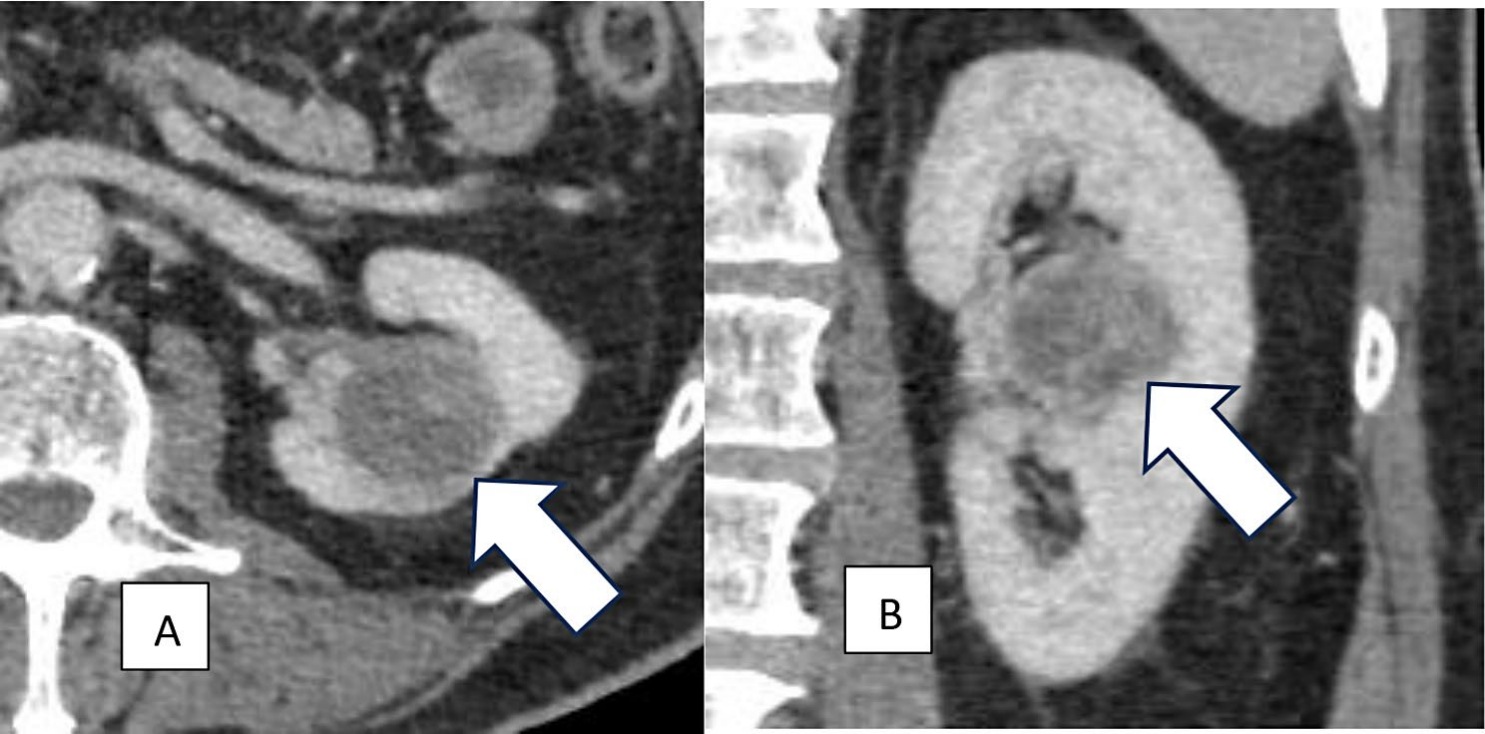
(**A**): Contrast-enhanced axial (**A**) and coronal CT images (**B**) of the abdomen at the level of the left kidney show an endophytic, circumscribed renal mass. While renal sinus invasion can be subtle and often challenging to detect on CT scan (such as in this case), pathology results are essential for accurate diagnosis and staging. Pathology results in that case were positive for clear cell RCC with renal sinus involvement, representing pT3a disease

**Fig. 3:**
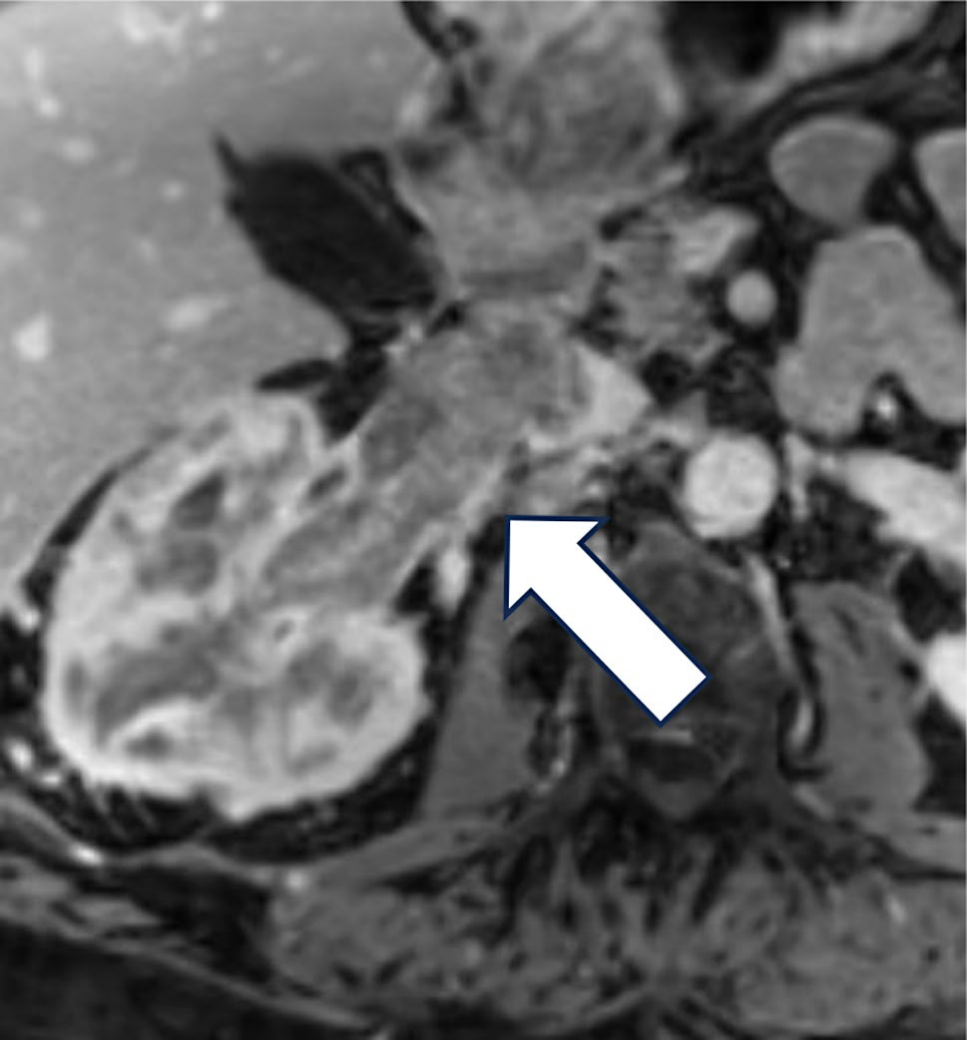
Axial 3D MR post contrast images show an extensive, heterogeneous right renal mass infiltrating the renal sinus, with tumor thrombus involvement of the renal vein (RV) and inferior vena cava (IVC). Pathology results were positive for high-grade chromophobe RCC subtype, negative surgical margins and renal vein tumor thrombus extending into the IVC

Radiologic identification of tumor invasion of critical anatomic structures such as renal sinus fat, perinephric fat, and venous system can be challenging, which may lead to potential understaging preoperatively [22] (Figs. 2 and 3). There is suboptimal CT sensitivity (59–88%) and specificity (71–93%) for Stage T3a disease, and pre-operative detection of T3a disease could influence the surgical approach [23]. Recently, several AI-based models have been developed for the prediction of locally advanced RCC using preoperative imaging. Yang et al. developed a radiomics model for the prediction of renal capsule invasion using preoperative computed tomography (CT) with different phases: unenhanced phases (UP), corticomedullary phases (CMP) and nephrographic phases (NP). The areas under the curves (AUCs) from the receiver operating characteristics (ROC) curve analysis were used to analyze the performance of the built models. The performance of the models on CMP exhibited the highest AUC (AUC = 0.81) compared to UP and NP, mostly because of the tumor morphologic characteristics exhibited on CMP [24]. Similarly, Liu et al. developed a radiomics model using contrast-enhanced CT scans for preoperative prediction of perinephric fat invasion on imaging. All patients had biopsy-proven evidence of locally advanced disease. Of the 8 built models, recorded AUCs ranged between 0.783 and 0.926, with one outperforming radiologists [25]. Moreover, Zhao et al. developed a combined ML and radiomics based model for the prediction of venous wall invasion using preoperative CT enhanced images. Their model was able to detect venous wall invasion with AUC of 0.853 [26]. If locally advanced RCC features could be reliably predicted pre-operatively, this could enhance the potential role of neoadjuvant treatment options and interventions (stereotactic body radiotherapy or systemic therapies), which could improve outcomes.

### Prediction of synchronous distant metastases and cytoreductive nephrectomy considerations

It is estimated that up to 30% of RCC patients present with synchronous distant metastases (SDM) i.e., distant metastatic disease at the time of initial diagnosis. Although metastatic renal cell carcinoma (mRCC) is associated with a poor prognosis, early identification of SDM and strategic treatment-planning based on the volume and distribution of disease may improve patients’ survival [27–29]. Tumor size has long served as a strong predictor of SDM; tumors greater than 3 cm in diameter have been associated with a higher risk for SDM compared to smaller ones [30–32]. There is increasing awareness that small RCCs are often indolent and may not need surgical management. While metastatic disease from small RCCs is exceedingly rare, it would be helpful to better risk-stratify these masses [33, 34]. The availability of more sophisticated imaging techniques has contributed to increased detection of SDM. However, the chance of missing or misdiagnosis of SDM still exists, partly because of small lesion size and/or atypical radiologic appearance [35–37].

Recent studies have used radiomics to build models for the prediction of SDM (Table 2) [38–40]. Bai et al. are among the first to construct a multiparametric magnetic resonance imaging (MRI) radiomic- based model combining multiple clinicoradiologic parameters for the prediction of SDM in patients with clear cell RCC (ccRCC). The model was able to predict SDM in the training and validation cohorts with an area under the curve (AUC) > 0.800. Moreover, the developed nomogram was able to equally predict SDM in relatively smaller tumors (< 4 cm) with an AUC = 0.875 and in larger tumors (size > 10 cm) with an AUC = 0.881 [38]. Wen et al. have designed a CT radiomic model instead for individualized prediction of SDM in patients with ccRCC. Their radiomic model was able to achieve similar results as Bai et al. in both training and validation cohorts with an AUC > 0.800 [39]. In another report, Yu et al. highlighted the importance of incorporating both CT-derived radiomic features with clinical parameters for enhanced detection of SDM. They developed a novel CT radiomic nomogram combining radiomics signature with clinical parameters and compared the performance of the CT radiomic nomogram to the individual performance of the radiomic signature and clinical-derived models separately. The CT radiomic model (combining both radiomic and clinical data) outperformed both radiomic signature and clinical models with AUC > 0.900 in both internal and external validation cohorts [40]. AI has the potential to enhance the detection of SDM using traditional imaging techniques, and risk-based imaging protocols could be explored to detect the presence of SDM most accurately.

**Table 2:** This table summarizes the models developed for the prediction of SDM, their inputs and performance

	Model	Input	Performance	Limitations
Bai et al.	MRI radiomics-based nomogram for the prediction of SDM	Radiomic-score and SDM-related clinic-radiologic characteristics in 201 patients	-Training: 0.914 -Internal validation: 0.854 -External validation: 0.816	-Retrospective study -Some SDM not pathologically proven -normogram developed using 3T, contrast-enhanced MRI - No real-world validation
Wen et al.	Radiomics model for preoperative prediction of SDM in ccRCC patients.	Quantitative extraction of shape, size and texture-based features in contrast-enhanced CT scan imaging of 172 subjects from The Cancer Imaging Archive (TCIA)	-Training: 0.890 -Internal validation: 0.830 -External validation: -	- Retrospective study - No external validation cohort - No real-world validation
Yu et al.	Radiomics model for the prediction of SDM in ccRCC	Contrast-enhanced CT scan imaging & clinicopathologic data in 242 patients	-Training: 0.882 -Internal validation: 0.916 -External validation: 0.925	- Retrospective study - Imbalance between study cohorts -CT acquisition parameters inconsistent -Some SDM not pathologically proven - No real-world validation

If a patient is diagnosed with renal cell carcinoma and SDM, cytoreductive nephrectomy (CN) remains a treatment option. CN refers to surgical removal of the kidney and primary tumor in a patient with metastases. It can be used to delay systemic therapy for patients who may be otherwise appropriate for active surveillance, a common management strategy for patients with indolent mRCC. CN can also be used as an adjunct to systemic therapy [41]. Recently, neoadjuvant immune-checkpoint based therapies were shown to enhance pathologic necrosis and systemic disease control as well as decrease tumor size prior to deferred cytoreductive nephrectomy, though surgical and survival outcomes were not altered [42]. It is hypothesized that CN may cause alterations in the humoral interaction between the primary tumor and distant metastases leading to a decrease in the tumor resistance and metastatic potential through the excision of the primary tumor [41]. However, role of cytoreductive nephrectomy in the immunotherapy era remains incompletely defined.

Prognostic prediction models have recently gained popularity to risk-stratify patients for specific treatment strategies including CN and systemic therapy. Machine learning-based models can screen patients and identify those who may respond well or poorly to a particular treatment by integrating complex connections between clinical features and patients’ outcomes. For instance, Yang et al. developed a ML-based prognostic model for patients undergoing CN and systemic therapy using seven clinical features including pathologic grade, T stage, N stage, number of metastatic sites, the presence/absence of brain or liver metastases, and whether patients’ have undergone metastasectomy as input. In a large population of greater than 900 subjects, patients with lower tumor grade, earlier tumor stage, no lymph node metastases, fewer metastatic sites, and those who underwent metastasectomy exhibited better prognosis after CN and systemic therapy. Conversely, the developed model identified roughly 15% of patients with metastatic ccRCC with poor 5-year survival after CN and systemic therapy, thus creating the potential for individualized treatment planning in this context [43].

### Prediction of recurrent and metastatic disease after resection of RCC

**Fig. 4:**
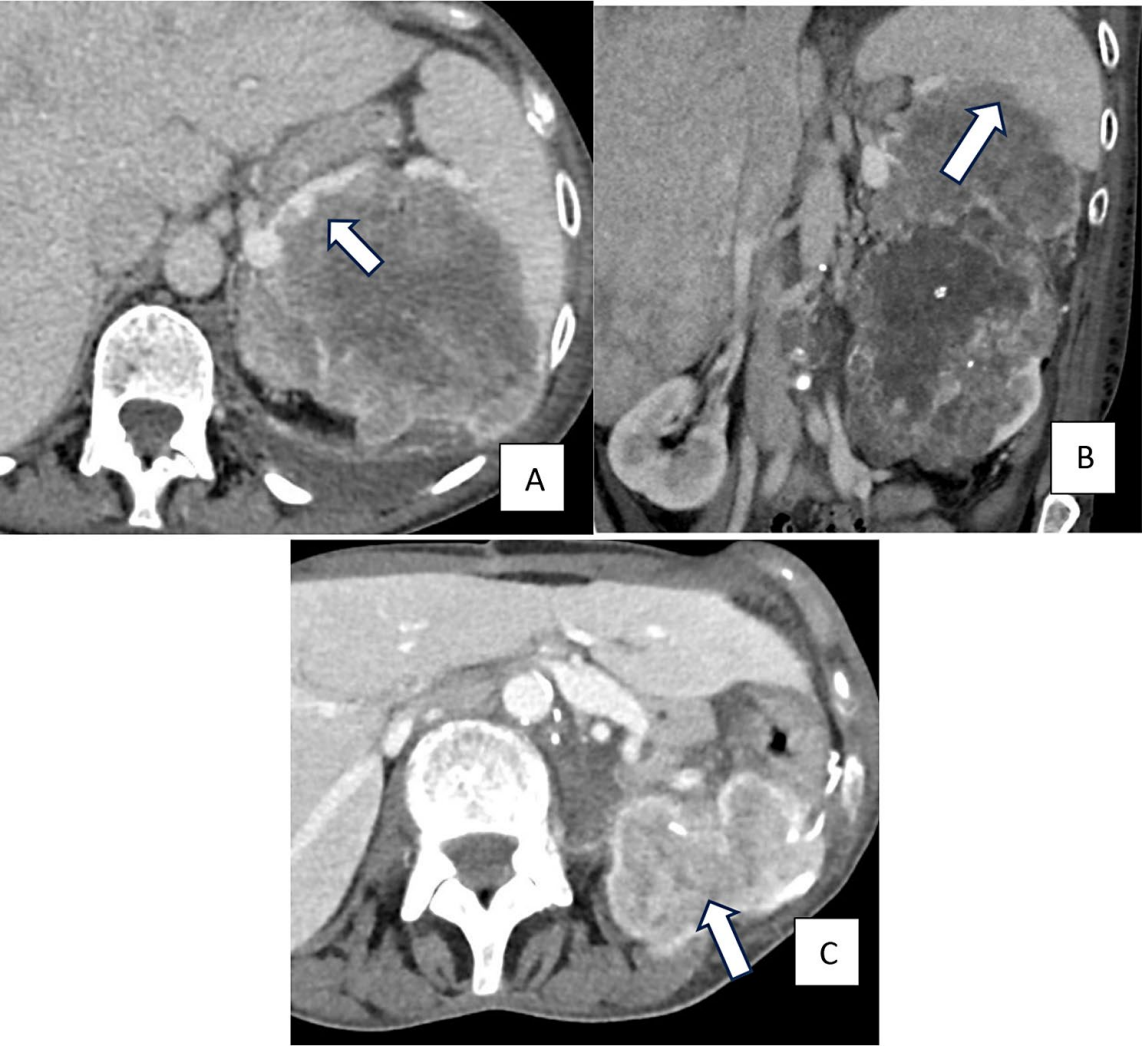
Contrast-enhanced axial (**A**) and coronal CT images (**B**) of the abdomen at the level of the left kidney show a large heterogeneous mass with extensive sarcomatoid differentiation invading the left kidney, left adrenal gland, distal pancreas and spleen. (**C**) Post-operative contrast-enhanced axial CT of the abdomen at the level of the left kidney shows local recurrence in the surgical bed

Surgical resection remains the gold standard treatment for localized RCC. Despite surgical resection, up to 30% of patients eventually develop recurrent or metastatic disease post-nephrectomy. Clinical nomograms have been created to identify high-risk patients for recurrence (tumor stage 2 with nuclear grade 4 or sarcomatoid differentiation, tumor stage 3 or higher, regional lymph node metastasis, or stage M1 with no evidence of disease) to help guide management decisions for adjuvant therapy, which has recently been shown to improve outcomes in high-risk patients [9] (Fig. 4).

Resected patients undergo imaging surveillance for recurrent or metastatic disease, which often occurs early (within 5 years post-nephrectomy) but can occur late (after 5 years post-nephrectomy). At present, the American Urologic Association, National Comprehensive Cancer Network, and European Association of Urology do not recommend imaging follow-up beyond 5 years. Nonetheless, up to one quarter of recurring patients exhibit late recurrence [44]. Risk-stratification of RCC patients has the potential to optimize clinical and imaging follow-up in those at highest risk for recurrent disease, to enhance detection of late recurrences while these may be amenable to local treatments. Kim et al. have developed one of the first ML-based models for the prediction of late recurrence post-nephrectomy using clinicopathologic data such as operation time and method, pathologic tumor and nodal stage, histology subtype and lymphovascular invasion [45]. Imaging-based datapoints were not incorporated. The highest performing model was able to successfully predict late recurrences with AUC = 0.740. Kim et al. demonstrated that operation type and method, node stage, and tumor size were significant variables in the prediction of late recurrence [45].

Imaging features have recently been incorporated into RCC recurrence prediction models [46, 47]. Denifell et al. have developed a radiomic-based model using post-operative clinico-pathological variables such as patients’ age, tumor size and grade with and without radiomics-derived data from pre-operative CT scans for the prediction of disease-free survival in patients with localized RCC. Decision curve analysis revealed better performance of the combined model vs. the clinical model for prediction of post-operative recurrence and subsequent need for adjuvant therapy. For instance, adopting ≥ 25% chance of disease recurrence within 5 years, the combined model would predict recurrence in 9 additional patients (per 1000) who would have been missed by the clinical model [46]. Zhao et al. have developed a radiomics metastasis score (RMS) for the prediction of metastasis in ccRCC linking pre-operative CT-derived radiomic features to major biological pathways involved in the pathogenesis of mRCC via RNA seq transcriptomics, including ECM-receptor interaction, focal adhesion, and PI3K-Akt pathways [47]. This study was among the first radiomic studies in ccRCC to examine biologically active processes, as opposed to individual underlying genetic mutations, of CT radiomics features associated with metastatic disease and outcomes.

More recently, Yang et al. developed a ML-based model that integrates multimodal data, including clinical, radiological (CT and ultrasound), and pathological parameters, to predict the risk of metastasis in ccRCC. Clinical inputs included demographic data (age, sex) and tumor-related factors such as TNM staging, histological grade, and the presence of comorbidities. Radiological features were extracted from both contrast-enhanced CT scans and ultrasound images, which provided tumor size, location, shape, and echogenicity, along with radiomic features like texture patterns (e.g., gray-level co-occurrence matrix, run-length matrix), shape descriptors (e.g., volume, sphericity), and first-order statistical features (e.g., mean intensity, skewness). Using this multimodal dataset, machine learning algorithms were applied to train and validate a predictive model on separate cohorts. The results showed that the model achieved high predictive performance, with AUC exceeding 0.85 in both the training and validation sets, indicating that multimodal integration significantly enhanced prediction accuracy compared to single-modality models. The study concluded that this multimodal machine learning model could serve as a valuable tool for personalized patient management, aiding in risk stratification and informing treatment decisions in ccRCC [48].

## Systemic therapy considerations and response assessment

Multiple therapeutic options exist for mRCC patients, including surgery for oligometastatic disease, locoregional therapies such as ablation and stereotactic body radiotherapy in certain circumstances and a host of systemic treatments. Treatment selection is empirical but informed by multiple factors such as the volume and distribution of disease, and the histologic subtype of RCC. In systemic treatment, immune check point inhibitors (ICI) now constitute the backbone of frontline treatment in metastatic ccRCC. In fact, ICIs, in combination with either a second immunomodulating antibody or a Vascular Endothelial Growth Factor (VEGF) targeted therapy, have become the treatment of choice for most treatment-naïve patients [49]. Patients typically continue a treatment until limiting toxicities develop or disease progression is confirmed using conventional response assessment (RECIST v1.1), and patients subsequently switch to an alternative treatment. There is limited AI data to predict the effectiveness of systemic therapies prior to treatment and during treatment, predominantly in small cohorts (Table 3).

**Table 3:** This table summarizes the models developed for the prediction of patients’ response to treatment, their inputs and performance

	Model	Input	Performance/Findings	Limitations
Rossi et al.	CT-based radiomic analysis	Radiomic features from CT scans of 53 mRCC patients	Correlated radiomic features with progression as the best response to ICI therapy	- Small sample size -Retrospective study - No validation cohorts
Park et al.	Clinical-CT texture models	Baseline and follow-up CT texture data combined with clinical data in 129 patients	Combined model predicted overall survival (C-index 0.7) and progression-free survival (C-index 0.63), outperforming clinical data alone	- Small sample size -Retrospective study - No validation cohorts -ROI determined by only one radiologist
Khene et al.	Texture analysis for survival prediction	Pre-treatment CT texture features in 48 patients	Identified predictors of overall and progression-free survival in mRCC patients treated with nivolumab.	- Small sample size -Retrospective study - No validation cohorts -Manual ROIs -Variable CT techniques
Neutrophil to Lymphocyte Ratio Studies	Biomarker analysis	Neutrophil to lymphocyte ratio	Low splenic volume 3 months after ICI treatment linked to improved overall survival	-Retrospective study - No validation cohorts
Splenic Volume Studies	Automated splenic segmentation	Splenic volume change measured using AI tools	Significant survival improvement associated with low splenic volume	- Small cohorts - Mixed treatment regimens - No validation cohorts
Negreros-Osuna et al.	CT-based radiomic model for TKI response prediction	Texture analysis of primary tumors and clinical data in 62 patients	Combined radiomic and clinical model outperformed models using radiomic or clinical data alone.	- Small sample size -Retrospective study - No validation cohorts
Chen et al.	CT-based radiomic model for short-term lesion response prediction	Radiomic features from baseline arterial phase (AP) and non-contrast (NC) CT scans in 36 patients with recurrent or mRCC	Delta feature-based model effectively predicted short-term lesion response to first-line TKIs in a small cohort. 0.940 (95% CI, 0.890‒0.990) in the training cohort and 0.916 (95% CI, 0.828‒1.000) in the validation cohort	-Small sample size -Retrospective study
Udayakumar et al.	Radiogenomic model using DCE-MRI	DCE-MRI, histopathology, and transcriptome correlatives in 49 ccRCC patients	High arterial spin labeling MRI correlated with favorable response to antiangiogenic regimens	-Small sample size - Cohort included mostly small tumors -Colocalization between presurgical imaging and postsurgical histologic analysis

### Prediction of patients; response of immune check point inhibitors

Texture analysis, an imaging processing method used to quantitatively analyze imaging-based spatial composition of lesions that may not be perceptible to the human eye, has been explored as a method for predicting response to therapy and outcomes in mRCC. Multiple studies have identified radiomic parameters for assessing patients’ response to systemic treatment. For instance, Rossi et al. studied CT- based radiomic features correlating with progression as the best response to ICI therapy in mRCC patients [50]. If confirmed in larger validation cohorts, such information could be used to help select or deselect patients for particular therapy. Park et al. developed clinical-CT texture models for the prediction of overall survival and progression-free survival of mRCC patients treated with ICI. CT-texture analysis was performed on both pre-treatment and first follow-up scans. Baseline and post treatment texture models could distinguish longer and shorter term overall survival, and the combined texture and clinical data model was able to better predict the overall survival (C-index, 0.7 vs. 0.63) and progression-free survival (C-index, 0.63 vs. 0.55) compared to the one using clinical data only [51]. Khene et al. also identified texture predictors of overall and progression-free survival in mRCC patients treated with nivolumab on pre-treatment CT [52].

In view of the absence of validated biomarkers for the prediction of patients’ response to immunotherapy in the setting of advanced RCC, researchers have studied the potential role of neutrophil to lymphocyte ratio as prognostic biomarker [53, 54]. More recently, change in splenic volume has been investigated as a surrogate marker for assessment of treatment response. Duwe et al. evaluated changes in spleen volume using automated splenic segmentation in patients with advanced RCC and urothelial cancer. In this small cohort, low splenic volume (lower 50% by median) three months after initiation of ICI treatment was associated with a significant improvement in overall survival. The study was limited by the retrospective nature and small cohort treated with multiple different agents [55].

### Prediction of patients’ response to tyrosine kinase inhibitors

CT texture analysis has also been explored in mRCC cohorts treated with tyrosine kinase inhibitors (TKI). Negreros-Osuna et al. developed CT- based radiomic models to predict response to tyrosine kinase inhibitors in mRCC patients using texture analysis of the primary tumors and clinical data. The model combing both radiomic and clinical data outperformed models developed from either radiomic or clinical data separately [56]. Chen et al. developed CT-based radiomic models to predict short-term lesion level response to TKIs in a small cohort of patients treated with first line targeted therapy. Radiomic features on baseline scans were extracted on arterial phase (AP) and non-contrast (NC) series, and the model based on delta features was concluded to predict short term response [57].

Udayakumar et al. have proposed a vertically integrated colocalization radiogenomic model using dynamic contrast-enhanced MRI (DCE-MRI) in the prediction of both tumoral angiogenic and inflammatory pathways while using histopathologic and transcriptome correlatives. The study included 49 patients with ccRCC (19 patients with mRCC on antiangiogenic or ICI first line treatment) who underwent DCI-MRI prior to nephrectomy. They have further corroborated that tumors with high baseline arterial spin labeled MRI, a technique that estimates tissue perfusion in ccRCC, often correlates with favorable response to antiangiogenic regimens in patients with mRCC [58]. Similar results were reported by Singla et al. in the assessment of contrast-enhancement in pancreatic metastases secondary to RCC, whereby these metastases exhibited intense contrast-enhancement and a favorable response to treatment to antiangiogenic treatment but refractoriness to ICI [59].

## Current challenges and limitations

The integration of AI in kidney cancer diagnosis offers significant potential but is hindered by numerous challenges related to data availability, quality, privacy, and algorithmic performance. High-quality and diverse datasets, such as multiphase CT and MRI images, are essential for building reliable AI models. However, insufficient data can result in underfitting or overfitting, where the model fails to generalize well to new data. Privacy regulations and ethical concerns further restrict access to sensitive medical data, limiting the development of robust AI systems [60]. On the other hand, new AI tools should be applied and rigorously tested in different populations for performance prior to clinical implementation, likely requiring industry and academic collaboration to fully develop.

The complexity of kidney cancer data—spanning imaging, genetic profiles, and clinical records—demands advanced algorithms capable of handling noise and extracting meaningful patterns. Differentiating between cancer subtypes is particularly challenging due to subtle variations in imaging and genomic data. Moreover, algorithms trained on specific datasets may fail to generalize across varying clinical settings, limiting their applicability in real-world scenarios, making it difficult for healthcare professionals to trust and implement new advances [60]. Reproducibility studies are needed for external validation. Models should be incorporated into large prospective clinical trials where possible.

Concerns over privacy and security create significant barriers. The sensitive nature of medical data raises risks of breaches and cyberattacks, potentially exposing patients’ private information and fueling discrimination. Robust cybersecurity measures, transparent data governance, and improved de-identification techniques are essential. Empowering patients and ensuring ethical handling of data will be critical to building trust and maximizing the potential of AI in kidney cancer diagnosis and management. Only by navigating these obstacles can AI achieve accurate and equitable diagnostic solutions while safeguarding patient privacy [60].

## Future directions

In general, this review has highlighted the potential role of AI in the complex care in patients with locally advanced and metastatic RCC. It has also shed light on the importance of combining clinicopathological, molecular and multiphasic imaging data to develop and validate AI-based solutions in the realm of advanced RCC.

AI models can move beyond the realm of research to clinical implementation, to aid in management decisions by analyzing patients’ clinicopathological and radiological data, as models combining both datasets have demonstrated superior performance compared to those relying solely on one of the two. While the implementation of these models into clinical practice is often a multistep process requiring a holistic approach addressing technical, workflow, and organizational challenges, the adoption of AI models into oncologic care, including radiology, is inevitable. A fundamental first step is the integration of formal AI education and workshops into clinical training and radiology faculty development. This ensures that healthcare providers acquire the necessary skills to critically evaluate AI models and validate their outcomes, paving the way for more informed and effective clinical implementation. While AI is poised to significantly enhance the clinical management of advanced RCC in several key areas, future studies should compare the performance of radiologists vs. their performance when provided with these models. Assuming a significant increase in their performance (higher accuracy and turnover time), implementing AI algorithms in RCC preoperative staging can assist radiologists accurately identify risk features, often associated with poorer prognosis and essential for determining optimal management strategies.

Multiphase CT and MRI are essential tools in renal tumor imaging, significantly shaping AI model development and clinical applications. While CMP remains vital for CT imaging of renal masses, multiparametric MRI provides additional benefits, particularly in identifying microscopic fat and subtle enhancement. Thus, the selection of disease-specific imaging protocols directly affects tumor visualization, characterization and data consistency, decreasing variability while improving accuracy of models. Future AI models should strive to improve their ability to characterize RCC on non-dedicated protocols, such as single-phase CT. While this endeavor is technically challenging, it holds significant clinical value since many tumors are incidentally detected on single phases studies, often necessitating re-scanning patients. Developing AI models capable of reliably interpreting such images would broaden their clinical applicability and enhance diagnostic accuracy in less-than-ideal scenarios but also decrease patients’ radiation exposure. AI may also be helpful identifying the most aggressive renal masses meriting biopsy or resection.

Approximately 30% of patients diagnosed with RCC will develop metachronous distant metastases (MDM) i.e., distant metastases after initial diagnosis, later in the course of the disease [61]. RCC metastases commonly affect the lungs, bones, liver, and adrenal glands, with site-specific prognostic differences [62]. AI has shown promise in predicting metastases in high-risk sites to enable earlier interventions. For example, machine learning (ML) models by Xu et al. [63] and Ji et al. [64] outperformed traditional nomograms in predicting bone involvement, with AUC values exceeding 0.85. To predict brain involvement, Kim et al. developed an ML model predicting brain metastases with moderate success (AUC = 0.716), limited by small sample size [65]. For liver metastases, Wang et al.’s XGB-based ML model achieved high accuracy (AUC = 0.947 [66]. However, these models are primarily based on clinical data, and integration of radiologic findings into future studies could enhance prediction accuracy and clinical application. Future studies incorporating radiologic data have the potential to enhance modeling to better predict MDM to various sites.

Traditional models like the MSKCC and IMDC (Heng score) stratify mRCC patients by risk groups, informing treatment plans. However, advancements in AI have introduced more precise prediction tools, aligning with National Comprehensive Cancer Network’s (NCCN) 2024 guidelines, which suggest that follow-up may be individualized based on surgical status, treatment schedules, side effects, comorbidities, and symptoms, thereby tailoring follow-up recommendations to each patient’s unique risk profile [67]. For instance, Buchner et al.‘s ANN demonstrated 95% accuracy in predicting overall survival, outperforming traditional models [68]. Similarly, Barken et al.‘s AI-based model surpassed MSKCC and IMDC for predicting 3- and 5-year survival, with high clinical utility [69]. These tools, however, require further validation, as they were developed retrospectively using limited clinical datasets. Furthermore, AI has significant potential to revolutionize RCC systemic treatment approach through leveraging imaging to assess the entire tumor burden non-invasively. RCC is well known for its pronounced tumor heterogeneity, both between primary and metastatic sites and among different metastases within the same patient. This variability complicates treatment selection, as different tumor regions may exhibit distinct molecular profiles and therapeutic responses [10]. By integrating AI with radiomics and deep learning models, clinicians may gain deeper insights into treatment resistance patterns and optimize therapy selection without the need for repeated invasive biopsies. However, this application remains in its early stages, as AI-based decision support for systemic therapy in RCC requires extensive validation through prospective studies. Given the complexity of RCC management, a multidisciplinary collaboration between radiologists, oncologists, data scientists, and pathologists will be essential to refine AI applications and align them with the most pressing clinical needs.

AI has the potential to play a pivotal role in the management of advanced and metastatic RCC. Models built using both clinicopathological and radiological data have demonstrated superior performance compared to those relying solely on one of the two. In time, such models can move beyond the realm of research to clinical implementation, to aid in management decisions over the care continuum, from the time of diagnosis, to post treatment surveillance and ultimately to therapy selection to optimize the care of patients with advanced or metastatic RCC.

## Data Availability

No datasets were generated or analysed during the current study.

## Abbreviations

AI: Artificial intelligence
ML: Machine learning
DL: Deep learning
NNs: Neural networks
ANNs: Artificial neural networks
CNNs: Convolutional neural networks
RNNs: Recurrent neural networks
NLPs: Natural language processing
RCC: Renal cell carcinoma
AJCC: American Joint Committee on Cancer
CT: Computed tomography
UP: Unenhanced phases
CMP: Corticomedullary phases
NP: Nephrographic phases
AUCs: Areas under the curves
ROC: Receiver operating characteristics
SDM: Synchronous distant metastases
mRCC: Metastatic renal cell carcinoma
MRI: Magnetic resonance imaging
ccRCC: Clear cell RCC
CN: Cytoreductive nephrectomy
RMS: Radiomics metastasis score
ICI: Immune check point inhibitors
VEGF: Vascular Endothelial Growth Factor
TKI: Tyrosine kinase inhibitors
AP: Arterial phase
NC: Non-contrast
DCE-MRI: Dynamic contrast-enhanced MRI
MDM: Metachronous distant metastases
NCCN: National Comprehensive Cancer Network’s
